# An additive manufacturing approach based on electrohydrodynamic printing to fabricate P3HT:PCBM thin films

**DOI:** 10.1038/s41598-023-43113-x

**Published:** 2023-09-28

**Authors:** Zulfikre Esa, Malik Muhammad Nauman, Lei Jin, Muhammad Usman Khalid, Juliana Hj Zaini, Asif Iqbal, Kamran Ali, Brahim Aïssa, Federico Rosei

**Affiliations:** 1 Faculty of Integrated Technologies, Universiti Brunei Darussalam, Bandar Seri Begawan, BE 1410 Brunei Darussalam; 2 Centre for Energy, Materials and Telecommunications, Institut National de la Recherche Scientifique, 1650 Boul. Lionel-Boulet, Varennes, QC J3X 1P7 Canada; 3 College of Computer and Information Sciences, Imam Mohammad Ibn Saud Islamic University, 11564 Riyadh, Saudi Arabia; 4 College of Science and Engineering, Hamad Bin Khalifa University, Ar Rayyan, Qatar

**Keywords:** Materials for devices, Nanoscale materials, Engineering

## Abstract

Additive manufacturing (AM) enables the production of high value and high performance components with applications from aerospace to biomedical fields. We report here on the fabrication of poly(3-hexylthiophene): phenyl-C_61_-butyric acid methyl ester (P3HT:PCBM) thin films through the electrohydrodynamic atomization (EHDA) process and its integration as absorber layer for organic solar cells. Prior to the film fabrication, the optimization of the process was carried out by developing the operating envelope for the P3HT:PCBM ink to determine the optimal flow rate and the appropriate applied voltage to achieve a stable-cone deposition mode. The EHDA printed thin-film’s topography, morphology and optical properties were systematically analyzed. The root-mean-square roughness was found to vary significantly with the annealing temperature and the flow rate and ranged from 1.938 to 3.345 nm. The estimated film mass and thickness were found between 3.235 and 23.471 mg and 597.5 nm to 1.60 µm, respectively. The films exhibited a broad visible absorption spectrum ranging from ~ 340 to ~ 600 nm, with a maximum peak λ_max_ located at ~ 500 nm. As the annealing temperature and the flow rate were increased, discernible alterations in the PCBM clusters were consequently observed in the blends of the film and the size of the PCBM clusters has decreased by 3% while the distance between them was highly reduced by as much as 82%.

## Introduction

The technology of manufacturing highly efficient, lightweight, low-cost, and stable solar cells has progressed dramatically during the last two decades. One of the highly promising potential manufacturing candidates is the additive manufacturing (AM), which involves the layered production of objects. It is seen as a revolutionizing manufacturing owing to its unique ability to fabricate complex shapes and produce accurate systems^1^. Nowadays, human tissues, microelectromechanical systems (MEMS), biomaterials, food and much more are being produced with the help of AM. Among the different types of AM techniques currently in use in the industry^2^, micro/nanoscale 3D printing technologies such as electrohydrodynamic printing (EHDP), direct ink writing (DIW), two-photon lithography, and projection micro stereolithography (PμSL) have rapidly attracted the attention of researchers and have been studied in various areas, including solar cells^3^.

OSCs are classified according to their active layer architecture, i.e. bi-layer heterojunction and bulk-heterojunction (BHJ)^4^. Bi-layer heterojunction structures involve stacking donor and acceptor (D/A) materials on top of each other, whereas BHJ structures involve blending D/A materials to form the active layer of OSC. Bi-layer devices are limited by the D/A exciton diffusion length, where excitons are only generated at the interface of the two materials. In contrast, the active layer of BHJ structures contains both D/A materials blended together, providing greater flexibility for exciton diffusion^5^. Indeed, BHJ active layers have drawn increasing attention because of their multiple advantages such as high mechanical flexibility, light weight, semi-transparency and high-throughput fabrication^6,7^. Driven by the rapid development of organic photovoltaic materials and device engineering, OSCs have achieved power conversion efficiencies (PCEs) of over 18%^8^. Despite the great success in PCE enhancement, the practical applications of OSCs are limited by their low operational stability^9^. Indeed, the degradation of photoactive layer morphology under various environmental conditions (i.e., light, oxygen and heat) after long-term exposure remains a grand challenge for the commercialization of OSC technology^10^. More specifically, highly efficient OSCs require an optimized BHJ morphology with abundant electron donor/electron acceptor (D/A) interfaces for efficient exciton dissociation^11^. However, the intrinsic instability of the blend photoactive layer commonly occurs during prolonged illumination or thermal aging^12^.

In order to meet the requirement of high performance and stability, it is imperative to develop effective strategies to achieve a stable and efficient optimized BHJ morphology^13^. Although the photon-to-charge conversion process is significantly complex and involves several steps, such as exciton diffusion and dissociation, charge transfer and transport, and charge recombination and collection^14^, it is well known that the morphology of BHJ blend film plays a key role in all these processes^15^. Furthermore, the morphology is also tightly correlated with the device lifetime (i.e. stability) which is crucial for determining whether OSCs can get access into the consumer market^16^.

In this regard, great efforts have been particularly devoted to optimize the manufacturing process. Poly(3-hexylthiophene) (P3HT) and [6, 6]-phenyl C61 butyric acid methyl ester (PCBM) are two fundamental materials widely utilized in BHJ OSCs. P3HT is a conjugated polymer that functions as the electron donor, whereas PCBM is a fullerene-based polymer that acts as the electron acceptor^17–19^. The state-of-the-art conjugated polymer can significantly benefit in fabrication and processing of OSCs. Several wet-process coating methods can be used in the fabrication process of the polymer thin films, such as casting, spin-coating, blading, screen printing, inkjet printing, spray-coating and roll-to-roll technique^20–22^.

Spin coating is one of the most common approaches in research studies in solar cell fabrication, because of its capability to produce a thin film and wide range of materials, by fine-tuning of the parameters such as spin speed and spin time etc. This method can produce film ranging from a few nanometers (30 nm—PAni film^23^_,_ 40 nm—P3HT film and 30 nm—PCBM film^24^) to micrometers (1–1.5 μm AZ3312—photoresist film^25^). However, the effectiveness of the above techniques is limited due to various factors such as thickness output, vacuum usage, mask application, and material wastage^26^. A promising approach is electrohydrodynamic atomization (EHDA), which employs an electric field to generate charged droplets that can be sprayed onto a substrate in a controlled way. In addition, EHDA is highly sustainable and green process as it is carried out in ambient conditions (i.e. vacuum free) and uses minimal materials with virtually less material wastage^27,28^.

EHD process methods specifically the OSC field and photoelectric converters generally are considered premature processes. In this work, we aim to improve the EHD process and employed the EHDA process to fabricate an optimize the deposition of P3HT:PCBM inks for OSC active layer application. We assessed the compatibility and the jetting of P3HT and PCBM inks materials. We demonstrate that by varying the flow rate and the annealing temperature, significant changes in the mass, PCBM cluster size, the separation distance and the structure variations are triggered accordingly.

## Materials and methods

### Materials and ink preparation

**Figure 1:**
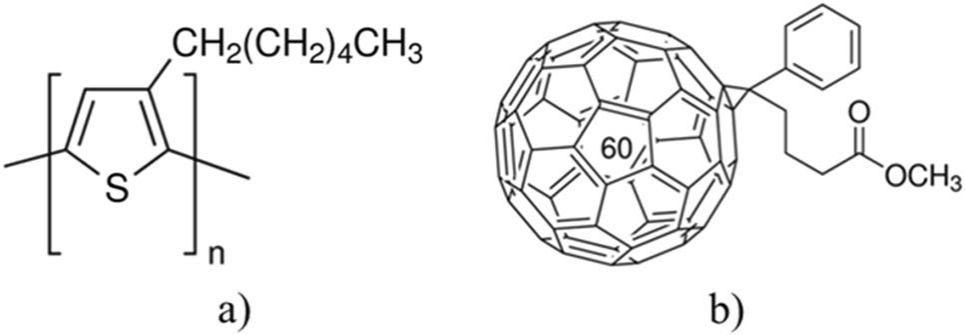
Schematic of the molecular structure of (**a**) P3HT; and (**b**) PCBM.

Regioregular P3HT (C_10_H_14_S)_n_ (average molecular weight M_w_ = 50–100 K, regioregularity = >90%, semiconductor properties = P-type (mobility = 1E−4 to 1E−1 cm^2^/V s) and PCBM (C_72_H_14_O_2_) (Molecular weight = 910.88 g/mol, assay = >99.5%, semiconductor properties = N-type (mobility = 0.21cm^2^/V s) were purchased from Sigma Aldrich. The molecular structure of P3HT and PCBM are shown in Figure 1. The inks were prepared by diluting 20 mg of P3HT and 20 mg of PCBM, in 1 ml of chlorobenzene (CB), separately, and stirred at room temperature at 500 rpm using a magnetic stirrer for 45 min. The two ink solutions were then mixed in a 1:1 ratio, and the prepared P3HT:PCBM inks in CB were put onto a shaker for 24 h before use. Corning Plain glass was used as a substrate, which was first cleaned ultrasonically with ethanol, di-water and acetone for 10 min respectively. Before transferring to different solvents, the substrate was oven-dried at 50 °C for 10 min.

### P3HT:PCBM and P3HT:PCBM deposition

**Figure 2:**
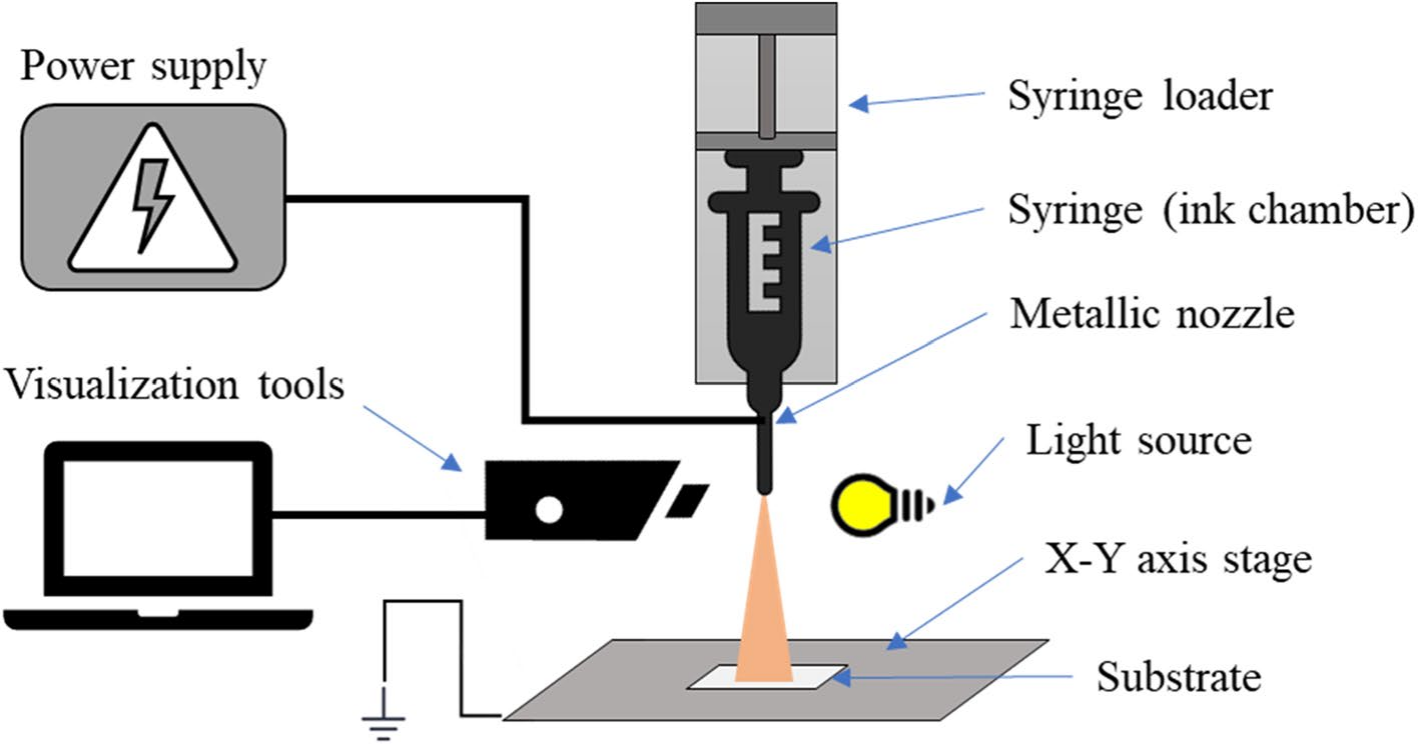
Schematic diagram of EHD printer setup.

To assess the printability of the developed inks via EHDA, the jetting produced by each individual ink, namely P3HT, PCBM, and P3HT: PCBM blend was analyzed. The schematic diagram of the fabrication setup is depicted in Fig. 2. An EHDA printer (NanoNC ESDR300HP) equipped with an ink supply system consisting of a syringe pump loader (eS-Pump: ESP200P), a syringe for storing ink (HENKE SASS WOLF, 10 ml (12 ml) NORM-EJECT) with a 27-gauge metallic nozzle from NanoNC is used. The inner and outer diameters of the metallic nozzle were 200 and 400 µm, respectively. Prior to the use, the metallic nozzle was cleaned with acetone in an ultrasonic bath for 30 min to remove any impurities. A high-voltage power supply connected to the metallic nozzle amplifies and creates an electrical force between the nozzle tip and the grounded substrate platform stage. A programmable stage was utilized to control the movement speed of the substrate and camera with a zoom lens to identify the cone-jet transition of the ink. The offset distance between the nozzle and substrate was kept at 20 mm and the flow rate of ink deposition was tested between 10 and 30 µl h^−1^, to identify the jetting interval.

P3HT:PCBM inks were sprayed onto a glass substrate via EHDA at a flow rate of 30 µl h^−1^ and 20 µl h^−1^, with a substrate movement speed of 20 mm h^−1^. The sprayed ink was printed in 40, 20, and 10 passes. The deposited materials were sintered in a muffle furnace at 150 ℃ and 175 ℃ for 15 min. Table 1 provides an overview of the printing parameters utilized in the production of the P3HT:PCBM thin films.

**Table 1:** Printing Parameters for EHD printed P3HT:PCBM thin films.

Layer/passes	Flow rate (µl h^−1^)	Sintering temperature (°C)	Label
20	30	150	PP1
20	30	175	PP2
20	20	150	PP3
20	20	175	PP4
40	30	150	PP5
40	20	175	PP6
40	30	175	PP7
10	30	150	PP8

### P3HT:PCBM thin film characterization

The root-mean-square roughness (Rq) and topography of EHDA-printed thin films were measured by atomic force microscopy (AFM, Enviroscope Veeco digital instrument), in a tapping mode, with a scanning size of 4 µm × 4 µm. Surface morphologies and cross-sectional images for thickness estimation, were characterized using scanning electron microscopy (SEM, Tescan vega3 LMH) operated at a very low accelerated voltage of 2 kV. The light absorbance and transmittance spectra of the P3HT:PCBM thin films were obtained with a UV–Vis spectrometer (Perkin Elmer LAMDA 650) in the 300–800 nm range. The deposited materials’ weight was measured using an analytical microbalance with a resolution of 1 mg.

ImageJ software was used to analyze and measure the PCBM patch size and distribution from the sample SEM images. PCBM sizes were measured by calculating the diameter of each patch. The average cluster size was measured by averaging multiple PCBM patches from the sample SEM image. While PCBM distribution distances were measured by averaging the distance between the PCBM patch with its neighbouring patches.

## Results and discussion

### P3HT, PCBM and P3HT:PCBM sprayability

**Figure 3:**
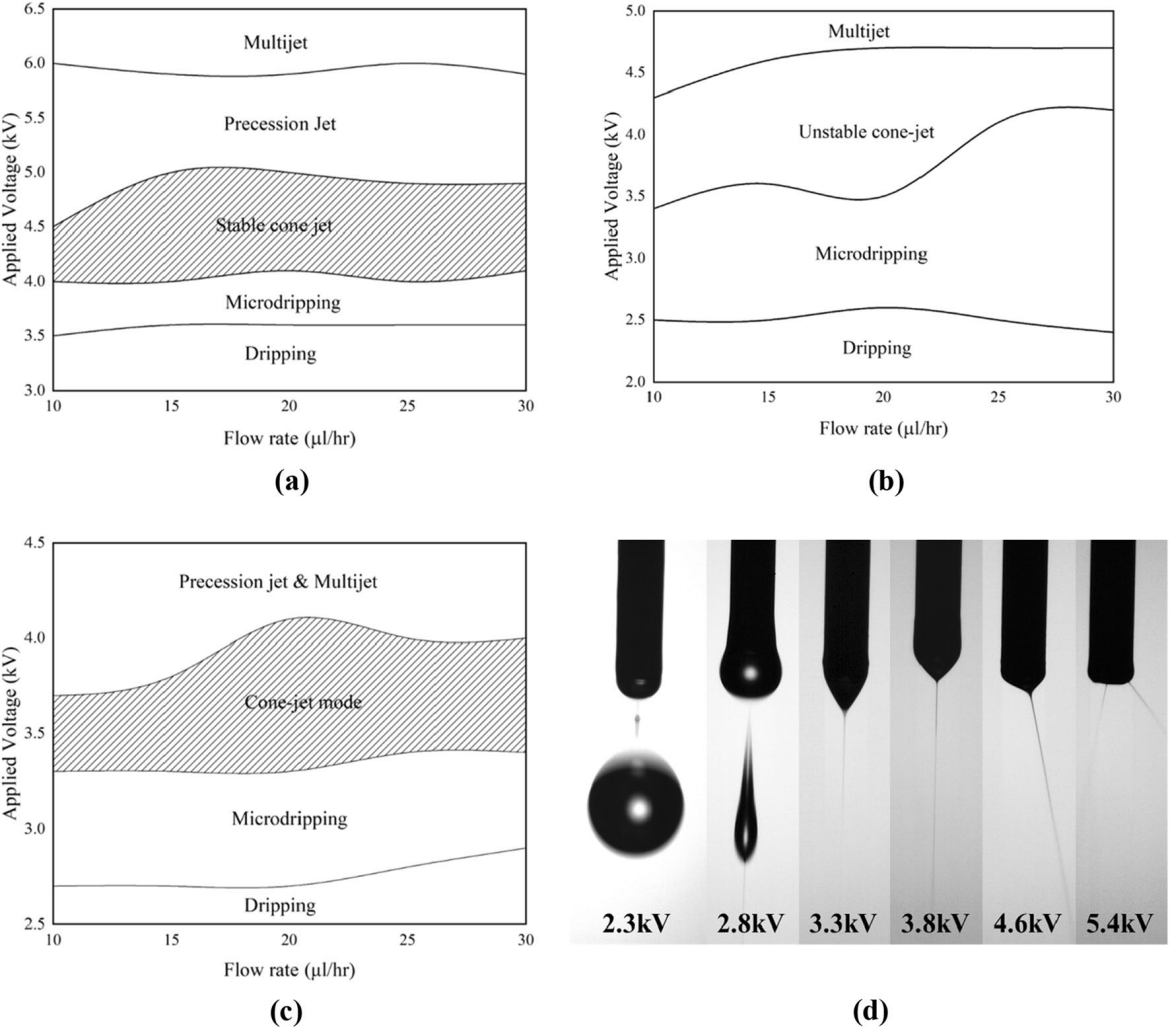
Operating enveloped of (**a**) P3HT ink; (**b**) PCBM ink; (**c**) P3HT:PCBM ink and (**d**) jetting profile dripping, microdripping, unstable cone-jet, stable cone-jet, precision jet, and multijet mode respectively.

To optimize the fabricating process and develop an operating envelope for P3HT, PCBM, and P3HT:PCBM films, EHDA was performed at different flow rates ranging from 10 to 30 µl/h, with increments of +5 µl/h (Fig. 3). Different jetting modes were observed at various flow rates and various applied voltages to the nozzle, including dripping, microdripping, cone-jet, unstable cone-jet, precession and multijet modes. Dripping mode occurs when the ink droplets are released from the nozzle with a diameter larger than the nozzle size. This mode was observed for P3HT (Fig. 3a), PCBM (Fig. 3b), and P3HT:PCBM (Fig. 3c) inks at voltage ranges of 0 to ~3.5 kV, 0 to ~2.5 kV and 0 to ~2.8 kV, respectively. Microdripping mode occurs where deposited droplets are smaller than the nozzle size. This mode was observed for P3HT, PCBM, and P3HT:PCBM inks at voltage ranges of 3.5 to 4.0 kV, 2.4 to ~ 4.0 kV, and 2.5 to 3.3 kV, respectively. Stable cone-jet is the ideal jetting mode for EHDA deposition and was observed for P3HT and P3HT:PCBM ink at voltage ranges of 4.0 to 4.9 kV and 3.3 to 4.0 kV, respectively. However, PCBM ink produced an unstable cone-jet, which alternated between cone-jet and microdripping mode, at voltage ranges of 3.5 to 4.5 kV. Precession jet and multijet were observed from the all three developed inks at voltage ranges of > 4.6 kV, > 4.5 kV and > 4.0 kV, respectively. The precession jet occurs when the meniscus starts to skew, while multijet is a phenomenon where multiple streams, at least two, emanate from the nozzle and the number of points increases with an increase in voltage. Figure 3d illustrates the jetting profile of the inks, dripping, microdripping, unstable-cone jet, stable cone-jet, precision jet and nultijet respectively.

After conducting a sprayability analysis on the inks, it was found that only P3HT ink (Fig. 3a) and P3HT:PCBM (Fig. 3c) ink were capable of generating a stable cone jet mode, which is the optimal mode for creating a thin film via EHDA deposition process. In contrast, PCBM ink (Fig. 3b) was unable to achieve the cone-jet mode. Consequently, the sprayability analysis indicates that the EHDA deposition method is the sole viable option for creating a bulk-heterojunction thin film, rather than utilizing bilayer deposition for organic solar cell applications.

### EHDA deposited P3HT:PCBM thin film

**Figure 4:**
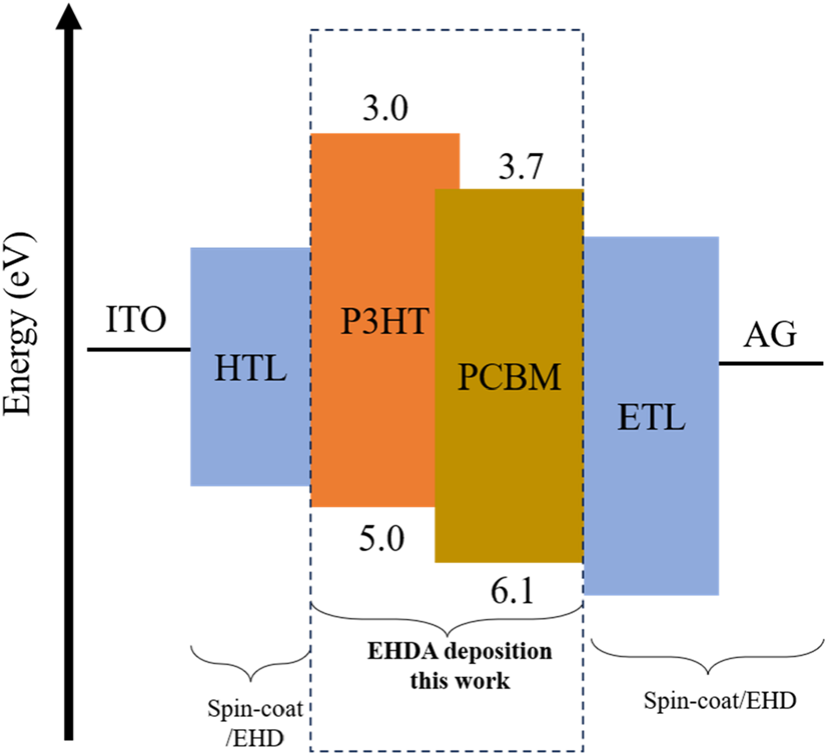
Energy levels alignment and the future proposed device architecture.

Figure 4, illustrates the energy level of P3HT:PCBM blend and proposed device architecture for future works. In this current work, we have utilized EHD printing to prepare the P3HT:PCBM blend thin film. The highest occupied molecular orbital (HOMO) of P3HT is typically higher than the HOMO of PCBM, and the lowest unoccupied molecular orbital (LUMO) of PCBM is generally lower than the LUMO of P3HT. As specified by the manufacturer HOMO and LUMO for P3HT, 5.0 eV and 3.0 eV respectively, and PCBM, 6.1 eV and 3.7 eV respectively.

**Figure 5:**
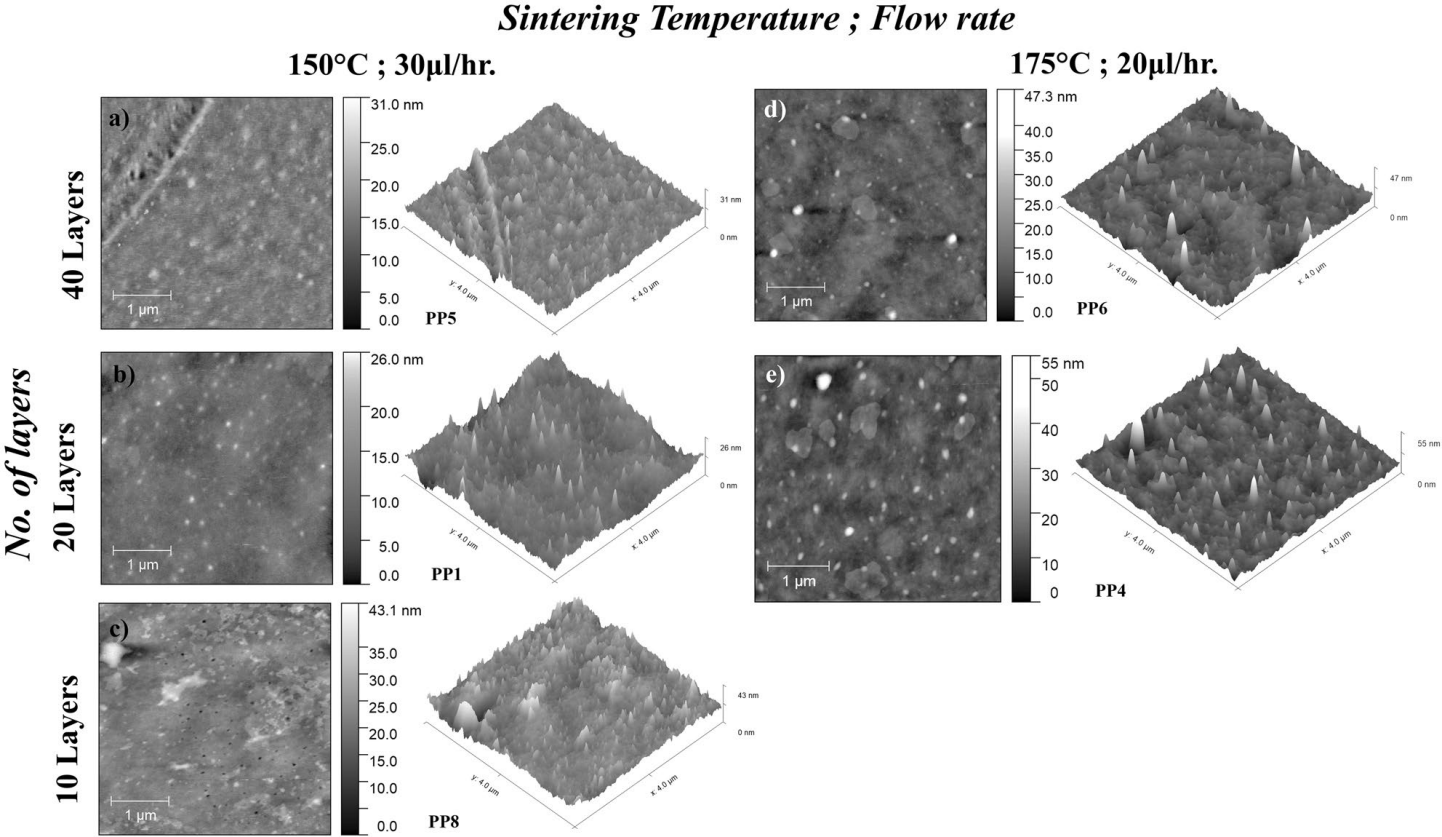
Tapping mode AFM images of EHD printed P3HT:PCBM thin film. (**a**) PP5; (**b**) PP1; (**c**) PP8; (**d**) PP6 and (**e**) PP4.

Figure 5 shows the AFM surface topography results for EHD-printed P3HT: PCBM films with varying printing parameters and annealing temperatures. Rq is used as a parameter to quantitatively characterize the surface roughness of the fabricated films. Table 2 shows that the films annealed at 150 °C (PP1, PP3, and PP5) exhibit lower Rq values-ranging from 1.938 to 2.352 nm, compared to those annealed at a higher temperature of 175 °C (PP2, PP4, PP6, and PP7), which show Rq values ranging from 2.416 to 3.910 nm. Thicker films annealed at lower temperature exhibit lower Rq values. However, the 10-layer film (PP8) annealed at 150 °C exhibits a higher Rq of 4.316 nm, which might be due to the presence of voids or pinholes in the deposited films caused by material’s evaporation during the printing or annealing process.

**Table 2:** Summarized EHD printed P3HT:PCBM thin film characterization results.

Sample	Rq (nm)	Ra (nm)	Avg. cluster size (µm^2^)	Avg. cluster distance (µm)	Weight (mg)	Avg. measured thickness (nm)
PP1	2.352	1.789	2.888	3.577	20.471	675.0
PP2	2.416	1.701	0.081	1.560	5.471	630.0
PP3	2.319	1.653	3.531	7.232	23.467	597.5
PP4	3.250	2.206	0.119	2.722	3.922	465.7
PP5	1.938	1.447	6.408	8.201	18.118	1600.0
PP6	3.022	2.206	0.133	3.502	9.470	654.3
PP7	3.910	3.016	0.079	2.255	9.573	1155.0
PP8	4.316	3.345	5.597	7.924	3.235	610.0

Furthermore, the comparison of fabricated films at flow rates of 30 and 20 µl/h (specifically, the PP1 & PP3, PP2 & PP4 and PP7 & PP6 samples) did not demonstrate a significant impact on the Rq (Rq was mainly affected by the number of printing passes and by the annealing temperature). EHDA printing flow rate has been found to have a significant effect on the distribution of the PCBM cluster, as demonstrated in Table 2. Specifically, at the same annealing temperature, the cluster average size was reduced by 82% (PP1 & PP3), 68% (PP2 & PP4) and 59% (PP7 & PP6).

**Figure 6:**
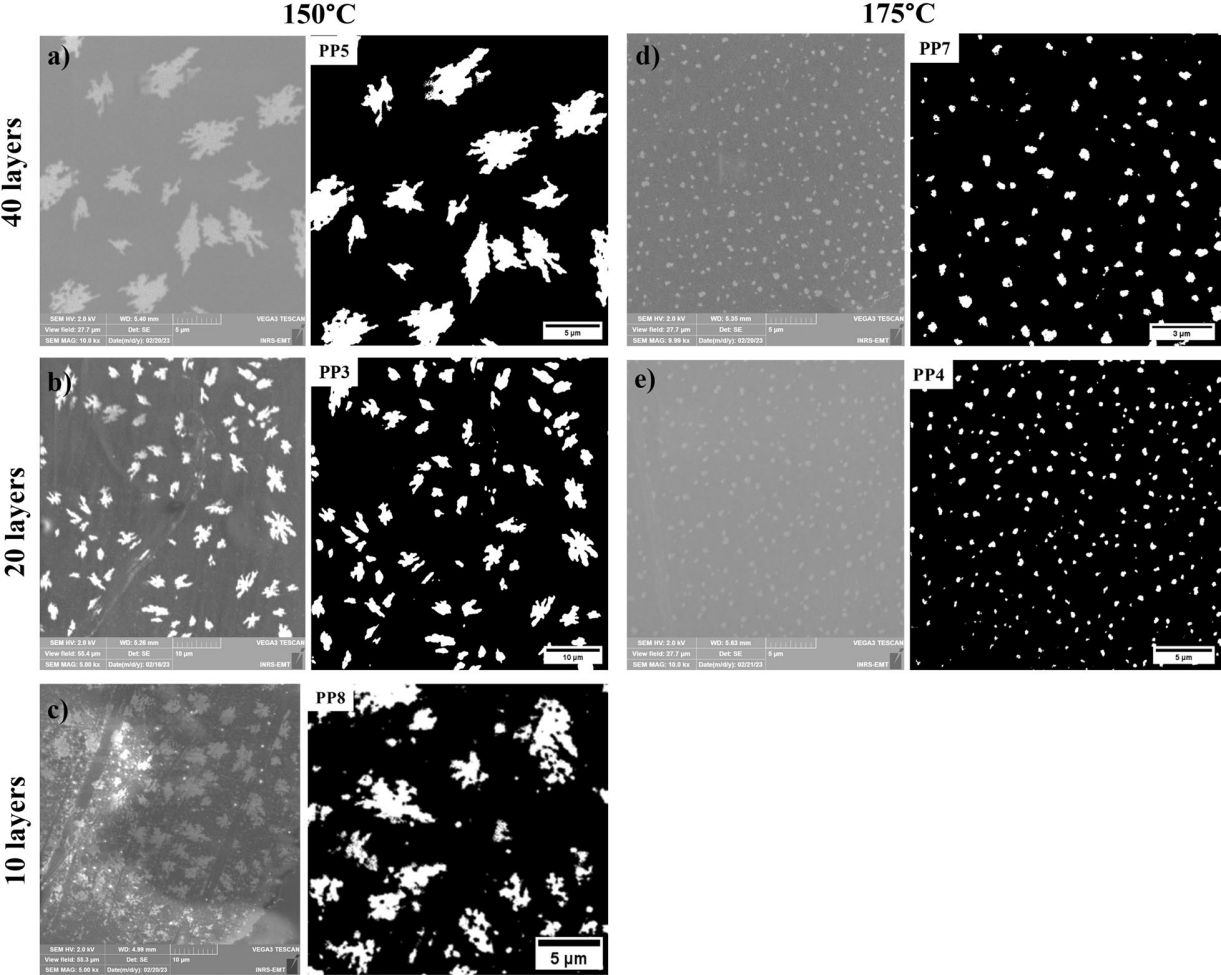
SEM and high contrast images of P3HT:PCBM film at different annealing temperatures and number of layers, (**a**) PP5; (**b**) PP3; (**c**) PP8; (**d**) PP7 and (**e**) PP4.

**Figure 7:**
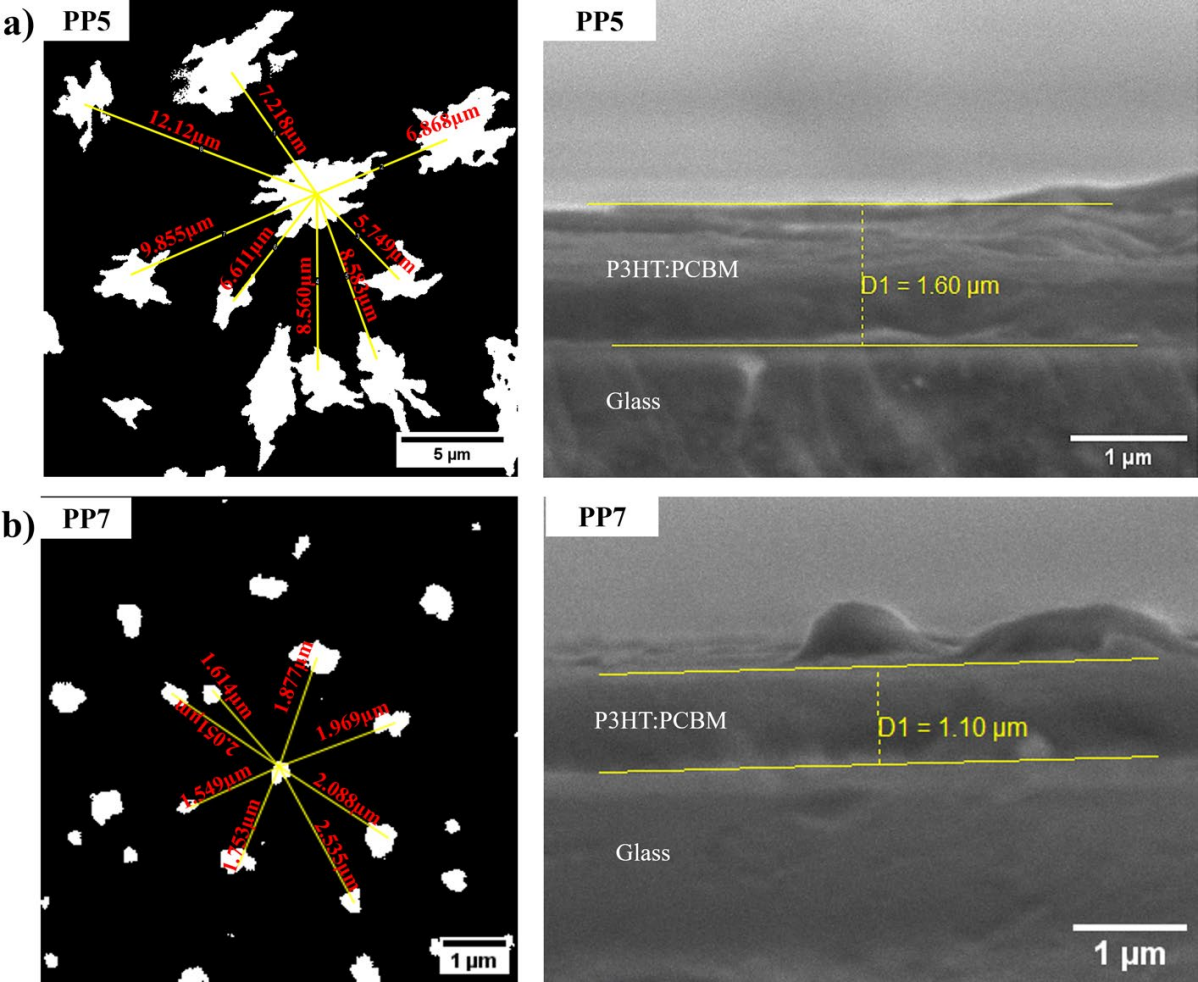
SEM high contrast image of cluster distributions (left) and cross-sectional image (right) (**a**) PP5 and (**b**) PP7.

The size of PCBM cluster distribution in the fabricated films is significantly reduced with higher annealing temperatures, as a result of PCBM segregation from P3HT^29^. The SEM images in Fig. 6 illustrates the impact of annealing temperature on EHDA-deposited PCBM. At lower annealing temperatures, PCBM clusters have an average size between 2.888 to 6.408 µm^2^, forming a flakes-like shape. However, at a higher annealing temperature, the size of the cluster was reduced by 82% and formed a circular shape. Figure 7a, b show the spatial distribution of the PCBM cluster as measured from the center of the cluster to its neighboring. Table 2 shows the results of the EHD printed thin films. Higher annealing temperature shows PCBM cluster has an average size of up to 0.13 µm^2^, results show the distribution of PCBM cluster approximately 1.50 to 3.60 µm. Whereas lower annealing temperature shows a large PCBM size of up to 6.40 µm^2^ and distributed approximately ~ 3.60 to 8.20 µm apart from other PCBM cluster patch. In sum, the distribution of the PCBM cluster is mainly resulting from the printing flow rate and annealing temperature. While high annealing process increased the Rq roughness of the developed film, and reduced the size of PCBM cluster.

Additionally, Table 2 shows the measured mass and calculated thickness of the thin films. Increasing the annealing temperature has resulted in a significant reduction in the film’ mass, due to its evaporation. A reduction in mass of 48%, 73%, and 83% associated to printing parameters of 30 µl h^−1^@20layers, 20 µl h^−1^@20layers and 30 µl h^−1^@40layers, respectively, was observed. This in turns has decreased the thickness of the film. The thickness measured from the side profile with PP5 films having the thickest film at 1.60 µm (40 layers), and PP3 films having the thinnest film at 597.5 nm (20 layers). The thickness of the film was impacted by the slow substrate movement speed and offset distance of the printing. It should be noted that printing parameters, including flow rate, substrate movement speed, offset distance, and annealing temperature, complement each other for producing a high quality thin film.

**Figure 8:**
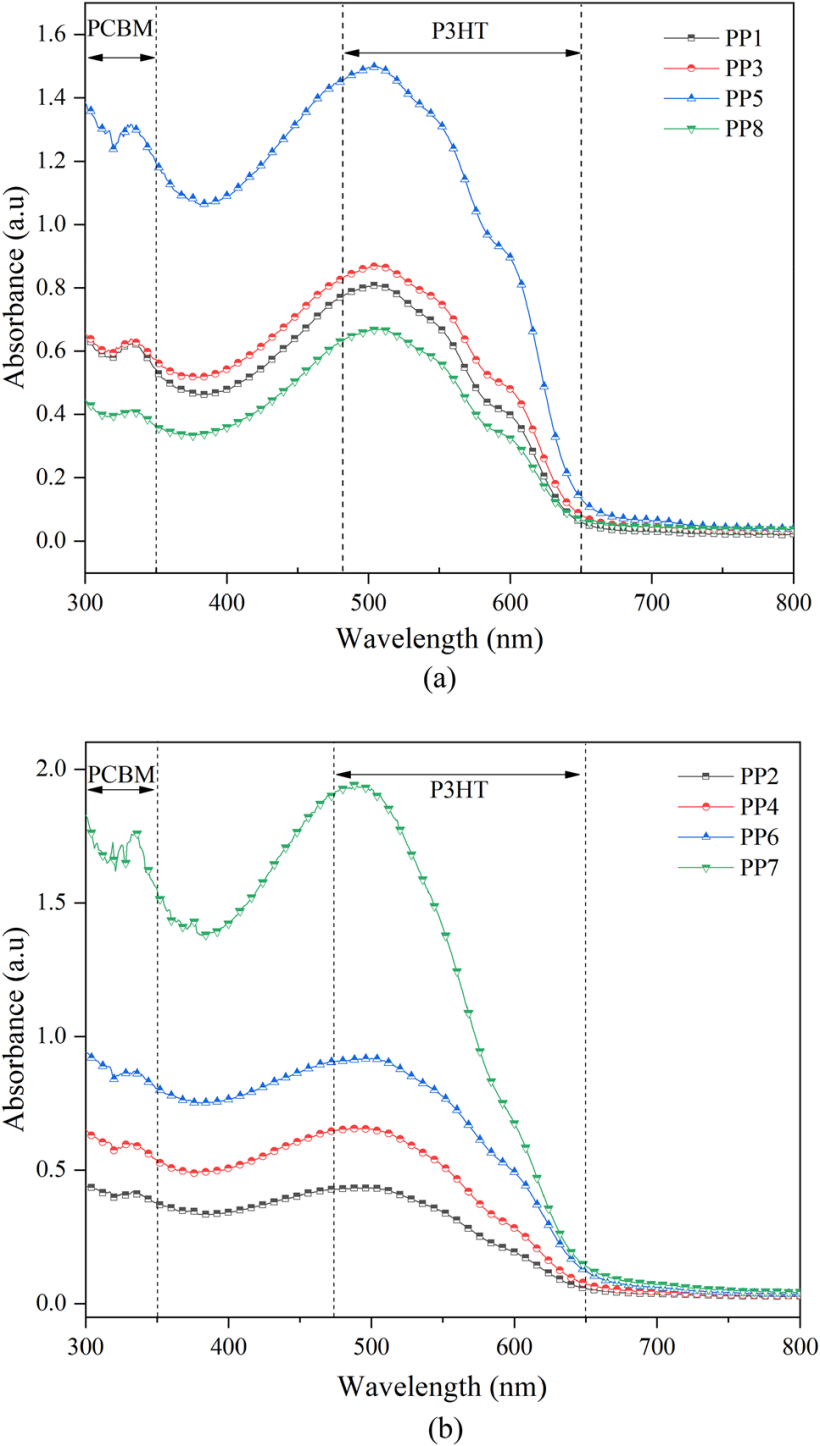
Absorption spectra of EHD printed P3HT:PCBM thin film with annealing temperature (**a**) 150 °C; and (**b**) 175 °C.

The absorption spectra of the EHD-printed P3HT:PCBM thin film at different parameter settings were then examined and results were displayed in Fig. 8a, b. Figure 8a illustrates the absorption spectra for film annealed at 150 °C and Fig. 8b shows the absorption spectra film annealed at 175 °C. The films were found to exhibit a broad visible spectral band spanning from 300 to 650 nm. In a typical PCBM film, there is an absorption peak located at 335 nm, and the primary absorption band of the P3HT material is located at 515 nm, with two shoulders at 558 nm and 609 nm^30^. The absorption of the films was proportional to the number of layer passes. The 40-pass films (PP5 and PP7) showed absorbance peaks (λ_max_) at 340 nm and broad features in the 500 nm region. When printed at a higher flow rate, the PP7 film has an absorbance peak at 340 nm and λ_max_ at ~ 480 nm. The PP5 film printed at a lower flow rate exhibited a good absorbance spectrum up to 600 nm, with a blueshift observed at ~ 320 nm and λ_max_ at ~ 520 nm. The λ_max_ of the PP5 film also showed a red shift and two shoulders located at ~ 590 and ~ 610 nm. Similar trends were found for the P3HT:PCBM films which were annealed at 150 °C. The presence of a slight peak shoulder indicated a strong interchain interaction between the P3HT chain and the PCBM in the blend^31^. Increasing the annealing temperature of the composite film resulted in red-shifted absorption spectra that matched the absorption spectrum of a P3HT film^29^. Results from UV–Vis in Fig. 8 and the study of the morphology of P3HT:PCBM thin film in Table 2 show a higher annealing post-process of the deposited materials increases the surface roughness of the thin film. A correlation effect between the surface roughness and the absorbance performance has been studied, it has shown higher surface roughness and non-uniform films help to increase the light absorption of the thin film with minimal reflection^32,33^. Previous researchers have reported that the distribution of P3HT and PCBM in the active layer can improve the power conversion efficiency of the OSCs. This can be achieved by shortening the path of electrons and holes transport between the cathode and the anode^34–36^.

## Conclusion

Additive manufacturing EHDA technique was developed to fabricate a P3HT:PCBM thin film for OSCs application. The optimized fabrication process allowed generating a stable cone jet under various operating conditions for P3HT:PCBM in a chlorobenzene ink. The effect of flow rates on the distribution of PCBM clusters in the P3HT:PCBM blends ink at different thicknesses and annealing temperatures was also investigated. The printing flow rate was observed to reduce the mean size of the PCBM cluster up to ~ 82% as the flow rate increased. The annealing temperature also played a critical role in the post-processing of the film in terms of Rq roughness measurement. Indeed, the EHDA thin film had an Rq roughness measured at ~ 1.938 to 4.316 nm, at different printing and post-process. The mass of the deposited P3HT:PCBM has decreased from ~ 50% to 83% with respect to the annealing temperature. The film thickness was measured at approximately 597.5 nm to 1.60 µm, depending on the printing parameters. The optical properties of the fabricated films show a broad absorption covering the entire visible region which is very relevant for OSC application. The peaks at 330 nm and λ_max_ ~ 500 nm, with two shoulders located at ~ 590 and 610 nm, indicated the absorption bands of PCBM and P3HT, respectively. Ongoing work is currently focusing on the EHDA fabrication of full OSCs and the effect of the PCBM clusters distribution in the photoactive layer on the device performance.

## Data Availability

The datasets used and/or analysed during the current study available from the corresponding author on reasonable request.

## References

[1] Prakash KS, Nancharaih T, Rao VVS (2018). Additive manufacturing techniques in manufacturing—An overview. Mater. Today Proc..

[2] Regassa Hunde B, Debebe Woldeyohannes A (2022). Future prospects of computer-aided design (CAD)—A review from the perspective of artificial intelligence (AI), extended reality, and 3D printing. Results Eng..

[3] Hunde BR, Woldeyohannes AD (2023). 3D printing and solar cell fabrication methods: A review of challenges, opportunities, and future prospects. Results Opt..

[4] Fukuda T (2016). Molecular ordering of spin-coated and electrosprayed P3HT:PCBM thin films and their applications to photovoltaic cell. Thin Solid Films.

[5] Ghorab M, Fattah A, Joodaki M (2022). Fundamentals of organic solar cells: A review on mobility issues and measurement methods. Optik (Stuttg).

[6] Liao C-Y (2020). Processing strategies for an organic photovoltaic module with over 10% efficiency. Joule.

[7] Zuo L (2022). Dilution effect for highly efficient multiple-component organic solar cells. Nat. Nanotechnol..

[8] Li C (2021). Non-fullerene acceptors with branched side chains and improved molecular packing to exceed 18% efficiency in organic solar cells. Nat. Energy.

[9] Bai Q (2022). Recent progress in low-cost noncovalently fused-ring electron acceptors for organic solar cells. Aggregate.

[10] Liao Q (2022). Highly stable organic solar cells based on an ultraviolet-resistant cathode interfacial layer. CCS Chem..

[11] Cheng H, Zhao Y, Yang Y (2022). Toward high-performance semitransparent organic photovoltaics with narrow-bandgap donors and non-fullerene acceptors. Adv. Energy Mater..

[12] Kim T, Choi J, Kim HJ, Lee W, Kim BJ (2017). Comparative study of thermal stability, morphology, and performance of all-polymer, fullerene-polymer, and ternary blend solar cells based on the same polymer donor. Macromolecules.

[13] Zhang Z (2019). Efficient and thermally stable organic solar cells based on small molecule donor and polymer acceptor. Nat. Commun..

[14] Schwarz KN (2020). Reduced recombination and capacitor-like charge buildup in an organic heterojunction. J. Am. Chem. Soc..

[15] Huang L (2019). Unraveling the morphology in solution-processed pseudo-bilayer planar heterojunction organic solar cells. ACS Appl. Mater. Interfaces.

[16] He Y (2019). Evidencing excellent thermal- and photostability for single-component organic solar cells with inherently built-in microstructure. Adv. Energy Mater..

[17] Helgesen M, Søndergaard R, Krebs FC (2010). Advanced materials and processes for polymer solar cell devices. J. Mater. Chem..

[18] Krebs FC, Espinosa N, Hösel M, Søndergaard RR, Jørgensen M (2014). 25th anniversary article: Rise to power—OPV-based solar parks. Adv. Mater..

[19] Jiang Y (2018). All electrospray printed perovskite solar cells. Nano Energy.

[20] Kumar A, Li G, Hong Z, Yang Y (2009). High efficiency polymer solar cells with vertically modulated nanoscale morphology. Nanotechnology.

[21] Ali K, Choi K-H, Muhammad NM (2014). Roll-to-roll atmospheric atomic layer deposition of Al_2_O_3_ thin films on PET substrates. Chem. Vap. Depos..

[22] Marinova N, Valero S, Delgado JL (2017). Organic and perovskite solar cells: Working principles, materials and interfaces. J Colloid Interface Sci.

[23] Zaidan KM, Hussein HF, Talib RA, Hassan AK (2011). Synthesis and characterization of (Pani/n-si)solar cell. Energy Proc..

[24] Lin X (2014). Morphological investigation of P3HT/PCBM heterojunction and its effects on the performance of bilayer organic solar cells. Synth. Met..

[25] Zhang, J. X. J. & Hoshino, K. Fundamentals of nano/microfabrication and scale effect. in *Molecular Sensors and Nanodevices*. 43–111 (Elsevier, 2019). 10.1016/B978-0-12-814862-4.00002-8.

[26] Mustafa HAM, Jameel DA (2021). Modeling and the main stages of spin coating process: A review. J. Appl. Sci. Technol. Trends.

[27] Choi KH, Muhammad NM, Rehmani MAA, Kim DS (2012). Hybrid piezo-electrostatic inkjet head for printed electronics. Proc. Inst. Mech. Eng. C J. Mech. Eng. Sci..

[28] Muhammad NM, Duraisamy N, Dang H-W, Jo J, Choi K-H (2012). Solution processed Al doped ZnO film fabrication through electrohydrodynamic atomization. Thin Solid Films.

[29] Sampaio PGV, González MOA (2022). A review on organic photovoltaic cell. Int. J. Energy Res..

[30] Vohra V, Razali NT, Wahi R, Ganzer L, Virgili T (2022). A comparative study of low-cost coating processes for green & sustainable organic solar cell active layer manufacturing. Opt. Mater. X.

[31] Xue R, Zhang J, Li Y, Li Y (2018). Organic solar cell materials toward commercialization. Small.

[32] Li G, Shrotriya V, Yao Y, Yang Y (2005). Investigation of annealing effects and film thickness dependence of polymer solar cells based on poly(3-hexylthiophene). J. Appl. Phys..

[33] Tan F (2016). Rough gold films as broadband absorbers for plasmonic enhancement of TiO_2_ photocurrent over 400–800 nm. Sci. Rep..

[34] Santhoshi Kiran KS, Preethi V, Kumar S (2022). A brief review of organic solar cells and materials involved in its fabrication. Mater. Today Proc..

[35] Shah SK, Abbas M, Ali M, Hirsch L, Gunnella R (2014). Optimal construction parameters of electrosprayed trilayer organic photovoltaic devices. J. Phys. D Appl. Phys..

[36] Singh I (2011). Effect of thermal annealing on the efficiency of poly (3-hexylthiphone):[6,6]-phenyl-C61-butyric acid methyl ester bulk heterojunction solar cells. J. Nanophoton..

